# Safe Abortion among Underprivileged Group Married Women of Low Resource Country: A Descriptive Cross-sectional Study

**DOI:** 10.31729/jnma.5298

**Published:** 2020-09-30

**Authors:** Sapana Budathoki, Prajita Mali, Rakshya Khadka, Bibek Rajbhandari

**Affiliations:** 1 Department of Public Health, Om Health Campus, Kathmandu, Nepal; 2 Centre for Mental Health and Counselling Service-Nepal, Nepal; 3 Nepal Police Hospital, Kathmandu, Nepal

**Keywords:** *abortion*, *Nepal*, *reproductive*

## Abstract

**Introduction:**

Unsafe Abortion is one of the leading causes of maternal death. The unhygienic and dangerous practice has been encountered in various geographical areas of Nepal. Despite its legalization, many women are still being not concerned and well informed regarding safe abortion and become victims of it. The main aim of this study was to assess the knowledge and practice regarding safe abortion among married women of reproductive (14 to 49) years of an underprivileged group of low resource country, Nepal.

**Methods:**

A descriptive cross-sectional study design was done in Rajbanshi community of Jhapa District. Data collection was done after taking ethical approval. Study population was selected conveniently. Data was collected by using a semi-structured questionnaire via face to face interviews among 420 married women of reproductive (14-49) years. All the extracted data were entered and analyzed using Statistical Package for the Social Services version 20. Descriptive analysis was done and presented using frequency and percentage.

**Results:**

Out of 420 respondents, 388 respondents (92.4%) found to have poor knowledge, regarding safe abortion. Likewise, only 44 respondents (10.05%) had practiced abortion, of which only 2 respondents (0.05%) had an unsafe abortion and 42 respondents (10%) had practiced safe abortion.

**Conclusions:**

Practices of unsafe abortion were prevalent. Respondents with poor knowledge were found to have done abortion. In this context, it can be concluded that knowledge regarding safe abortion can be increased by educating and providing awareness to the people of society.

## INTRODUCTION

World Health Organization (WHO) referred unsafe abortion as the unwise practice of terminating pregnancy by the untrained persons or in an inappropriate circumstances in the absence of minimum medical standards.^1^ Rajbanshi are one of the indigenous groups which are originally derived from the Koch in Bihar, Bengal, Asam of India and also Bangladesh, and they consider themselves as descendant of the Koch king in the late 15^th^ century.^2^ Since, they are agro-based laborious people with their own sets of language and culture, they are hesitant to change their lifestyles.^3^

Being economically, politically and socially backward in every sectors, along with cultural and social barriers there might not be adequate knowledge and proper access on sexual and reproductive health which increases high chances of conducting unsafe abortion in the Rajbanshi community.^4^

So, the study is aimed to assess the knowledge and practice regarding safeabortion among married women of reproductive (14 to 49) years of underprivileged group of low resource country, Nepal.

## METHODS

A descriptive cross-sectional study was conducted among 420 married women of reproductive (15-49) years in Ward no. 1, Bardashi Rural Municipality, Rajbanshi Community, Jhapa, Nepal.

Data was collected from January to February 2019. Ethical approval was taken from Nepal Health Research (Reg. no. 676/2018). Signed informed consent was taken from the participants. Study populations were married women of reproductive (15-49) years in Ward no. 1 , Bardashi Rural Municipality, Rajbanshi Community, Jhapa, Nepal. Married women of reproductive (15-49) years, who voluntarily willing to participate were included in the study. Women below and above the reproductive (15-49) years were excluded from the study.

The sample size was calculated using the following formula,

$$\begin{array}{ll} \text{n} & \text{= }{\text{Z}}^{\text{2}}\times \text{p}\times \text{q}/{\text{e}}^{\text{2}}\\ & \text{= }{\left(1.96\right)}^{\text{2}}\times (0.5)\times (1-0.5)/{\left(0.05\right)}^{\text{2}}\\ & \text{= }384 \end{array}$$

Where,

n = sample size
p = prevalence, 50%^5^
q = 1-p
Z= 1.96 for Confidence Interval of 95%
e= margin of error, 5%

Taking high non-response rate of 9%, the total sample size was 420. Systematic Random Sampling was done using the sampling frame of Baradashi Rural Municipality.

K^th^ item = 1559/420 = 3.7 ~ 4

The tool used for data collection in the study was semi structured questionnaireto assess the knowledge and practice towards safe abortion.

The questions were divided into 2 groups:

Part 1: Demographic information

Part 2: Questionnaire related to knowledge and practice regarding safe abortion.

For assessing level of knowledge, there are total 9 questions including 3 questions of multiple response. Likewise for assessing the practice, there are total 7 questions including 2 questions of multiple response. For the multiple response,the options which are correct, their mean value was calculated and recorded into variables i.e. poor knowledge and good knowledge. After computing the total score on knowledge a value range from 4 to 14 was obtained which was then added and divided by 2 for a mean value which was 8.5. Hence the value ranging from 4 to 8 was coded as poor knowledge and value ranging from 9 to 14 was coded as good knowledge.

The questionnaire in English was translated to the Nepali version for collecting data so that consistency was maintained. For reliability, data was pretested on 10% of the calculated sample size which was the representative study population other than the sample. Other additional editing to questionnairewas done according to the comments and result of pre-test which was found to have poor knowledge regarding safe abortion which was carried out in Jatrubari, Birtamode. The questionnaire was administered to the respondents via face to face interview with the approval through verbal and written consent with the help of mediator with the aim of minimizing language barrier.

Data were entered and analyzed in SPSS version 20.0. Descriptive analysis was done and presented using frequency and percentage.

## RESULTS

Out of 420 respondents, 388 respondents (92.4%) found to have poor knowledge, whereas 32 respondents (7.6%) had good knowledge regarding safe abortion. Likewise, the study revealed that among 420 respondents, only 44 respondents (10.05%) had practiced abortion, of which only 2 respondents (0.05%) had unsafe abortion and 42 respondents (10%) had practiced safe abortion.

Table 1 shows that among 420 respondents, majority are of age between 19-29 years i.e. 153 (36.4%). Similarly, 152 (36.2%) belongs to 30-40 years and 115 (27.4%) to 41-51 years. All the respondents wereHindu and mostly engaged in agriculture. Furthermore, 84 (20%), 90 (21.4%), and 23 (7.9%) were engaged in Foreign Employment, Daily Wages labor and Business respectively. More than half of the population are literate and near about two fifth of the population are illiterate. About 288 (68.6%) respondents are living in extended/joint family and 132 (31.4%) in nuclear family. About half of the population have monthly income of less than Rs.5000, and about one third of the population haveearnings between Rs.5000-10,000 and less than one fourth of the population have income more than Rs.10000.

**Table 1:** Socio-demographic characteristics (n = 420).

Variables	n (%)
**Age (in years)**	
19-29	153 (36.4)
30-40	152 (36.2)
41-51	115 (27.4)
**Religion**	
Hindu	420 (100)
**Family occupation**	
Daily wages labor	90 (21.4)
Agriculture	213 (50.7)
Foreign Employment	84 (20)
Business	33 (7.9)
**Education**	
Illiterate	167 (39.8)
Literate	253 (60.2)
**Type of Family**	
Nuclear	132 (31.4)
Extended/joint	288 (68.6)
**Monthly NRs**	
Less than 5000	1949 (46.2)
5000-10,000	137 (32.6)
>10000	89 (21.2)

Table 2 represents 304 (49.1%) of respondents have heard about abortion through peer discussion. Similarly only 36 (8.6%) know about the legalization of abortion service in Nepal. About 266 (63.3%) of respondents do not have knowledge about the complications of unsafe abortion. Those respondents who had knowledge 119 (56.7%) stated hemorrhage as the major complication of unsafe abortion. Overall Knowledge was found to be poor as out of 420 respondents, 388 (92.4%) had poor knowledge and only 32 (7.6%) had good knowledge about safe abortion.

**Table 2:** Knowledge about Safe Abortion (n= 420).

Factors related with knowledge	n (%)
**Heard about safe abortion**	
Yes	420 (100)
No	0 (0)
Source of Information^*^	
mass media	205 (33.1)
health facility	48 (7.8)
Exposure	40 (6.5)
Peer	304 (49.1)
School/colleges	22 (3.6)
**Safe abortion is done by**	
Health personnel in safe environment	420 (100)
Home/traditional healers	0 (0)
**Knowledge about legalization**	
No	384 (91.4)
Yes	36 (8.6)
**Appropriate time of abortion**	
12 weeks	36 (8.6)
Don’t know	384 (91.4)
**Condition for abortion**	
Mother had physical and mental disorder	8 (19.5)
Deformity in fetus	7 (17.1)
If pregnant women wants	26 (63.4)
Services available in Nepal^*^	
Government hospital	392 (65)
Private hospital	143 (23.7)
Safe abortion institution	68 (11.3)
**Know about complication**	
No	266 (63.3)
Yes	154 (36.7)
If yes, complications^*^	
Infection	15 (7.1)
Hemorrhage	119 (56.7)
perforation of uterus	18 (8.6)
Infertility	14 (6.7)
Death	44 (21)
**Total level of Knowledge**	
Poor knowledge(4-8)	388 (92.4)
Good knowledge(9-14)	32 (7.6)

* Multiple response

Table 3 shows that among 420 respondents, only 44 numbers (10.05%) of respondents have practiced abortion of which 2 had their abortion itself at home with the help of traditional healer. Majority of respondents i.e. 26 (59%) had their abortion at private hospital and about 33 (75%) had done abortion only to avoid unintended pregnancies. Most of the respondents reported to have faced hemorrhage as the major complication.

**Table 3:** Practice of Safe Abortion (n = 420).

Factors related to Practice	n (%)
Ever did abortion (420)	
No	376 (89.5)
Yes	Safe abortion 42 (10)
	Unsafe abortion 2 (0.05)
Place of abortion (n=44)	
Private Hospital	26 (59)
Government hospital	15 (34)
Safe abortion institution (Marie Stopes)	1 (2.45)
Home	2 (4.55)
Type of abortion	
Medical	33 (75)
Surgical	9 (20.45)
Home remedies (unsafe abortion)	2 (4.55)
Reason for conducting abortion	
Unwanted pregnancy	33 (75)
Complication	11 (25)
Complication after abortion (if yes)^*^	
Pain in lower abdomen	3 (15.8)
Heavy bleeding	11 (57.9)
Swelling of hand and legs	5 (26.3)
Family support received	
No	4 (9.1)
Yes	40 (90.9)
Type of family support received (if yes)^*^	
Financial	38 (55.1)
Transportation	16 (23.2)
Both	15 (21.7)

* Multiple response

## DISCUSSION

Several literatures have highlighted about knowledge being a conspicuousfactors in abortion seeking and decision making, especially for more youthful women.^5^ Thus, we conducted the study with the main aim of assessing the knowledge and practice about safe abortion among reproductive aged women residing in Rajbanshi community, Nepal.

The study showed that 92.4% of the women hadpoor knowledge regarding safe abortion. Similarly, study conducted in a group of 181 women, during their visit to Marie Stopes, Kathmandu, also revealed that the overall knowledge was low to moderate (43.6% to 46.4%), which suggests need for theinterventional program among reproductive age group of women about safe abortion.^6^ The study carried out in India to find out the effect of the educational intervention showed that there was significant difference of 61% between pre and post intervention which also supports that educational intervention was very effective in increasing awareness among the reproductive age of women and ultimately helpedin reducing mortality and morbidity related to abortion.^7^ Our study showed that about 75% of the women did abortion only because of the unintended pregnancies. This indicates that women had not practiced measures of contraception effectively. Enhancing access to the contraceptives and providing them various options of methods might reduce the rise in demand of abortions. Likewise,another study showed that 75% of those who had abortion was medical abortion, of which most of them had self-administered and faced to have hemorrhage as major complication which is similar to the study conducted in Sri Manakula Vinayagar Medical College and Hospital, which had found excessive bleeding as common presentation of which 77.5% in 12.5% faced from anemia and 5% of patients withshock.^8^ Our study also indicated that about 57.9% of the women who had undergone abortion, complained heavy bleeding as the complications of abortion. Thus, the preoperative identification of women with increased risk of hemorrhage along with prophylactic use of uterotonic medication and intraoperative ultrasound, could be one of the effective strategies for organized treatment and might remarkably reduce the practice of unsafe abortion.^9^

About 8.6% of the women had knowledge about the legalization of abortion in our study which was much lower than the study conducted in Armenia that showed 81% of knowledge among women about legalization of abortion.^10^

Thus, it can be said that capacity building, education and training programs are necessary with maximum participation of women from the indigenous and local community of Rajhbanshi. Similarly, massive and systematicawareness campaigns on national laws and policies regarding safe abortion helps women in exercising their rights and build up leadership and managerial skills. Elimination of traditional and superstitious practices harmful to women's health and ensuring access to gender sensitive health information by men and womenalong with quality and accessibility of family planning Information and Services to women are some of the important factors to be consider .

Our study has some limitations. First of all, Cross-sectional collection of data won't allow causal inferences to be drawn. Also, Rajbanshi has a diverse culture and it differs from place to place and particular area of Jhapa was selected as our setting for data collection so, findings cannot be generalized concerning whole country setting.

## CONCLUSIONS

Despite the legalization of abortion service, practices of unsafe abortion were found. Respondents with poor knowledge were found to have done abortion as compared to those who have good knowledge. Furthermore, knowledge regarding safe abortion can be increased by educating and providing awareness to the people of society and involving women in the decision making role so that the complications based on abortion and the unsafe practices of safe abortion can be reduced. Effective knowledge regarding contraceptive devices should be given along with the strict monitoring and supervision towards self-administration of abortion pills. Likewise support from the government and other concerned bodies regarding the implementation of mechanisms and effective measures to address the underlying traditional taboos and practices in Rajbanshi Community.

## References

[1] Ganatra B, Tunçalp Ö, Johnston HB, Johnson BR, Gülmezoglu AM, Temmerman M (2014). From concept to measurement: Operationalizing WHO's definition of unsafe abortion. Bull World Health Organ.

[2] Shrestha KK (2002). Ethnography of Jhapali Rajhbanshi. Nepal J Online.

[3] Subba N, Subba S (2014). Modification Of Delivery Practice In Rajbanshi Mothers Of Nepal. J Nobel Med Coll.

[4] Subba NR (2015). Traditional Practices on Mother and Child Health Care in Rajbanshi Traditional Practices on Mother and Child Health Care in Rajbanshi Community of Nepal. Am J Heal Res.

[5] Rogers C, Sapkota S, Paudel R, Dantas JAR (2019). Medical abortion in Nepal: A qualitative study on women's experiences at safe abortion services and pharmacies. Reprod Health.

[6] Khanal P, Sanjel K, Chalise HC (2014). Knowledge and practice of abortion among women in Nepal. Asia-Pacific E-Journal of Health Social Science.

[7] Kerns J, Steinauer J (2013). Management of postabortion hemorrhage. Contraception.

[8] Nivedita K, Shanthini F (2015). Is it safe to provide abortion pills over the counter? A study on outcome following self-medication with abortion pills. J Clin Diagnostic Res.

[9] Silwal K, Shrestha T, Dulal RK (2013). Effects of educational intervention among reproductive age group women on safe abortion. JNMA J Nepal Med Assoc.

[10] Chong E, Tsereteli T, Vardanyan S, Avagyan G, Winikoff B (2009). Knowledge, attitudes, and practice of abortion among women and doctors in Armenia. Eur J Contracept Reprod Heal Care.

